# Topological dynamics in spike-timing dependent plastic model neural networks

**DOI:** 10.3389/fncir.2013.00070

**Published:** 2013-04-18

**Authors:** David B. Stone, Claudia D. Tesche

**Affiliations:** Department of Psychology, University of New MexicoAlbuquerque, NM, USA

**Keywords:** spike-timing dependent plasticity (STDP), motif, topology, small-world, neural network, graph theory, simulation

## Abstract

Spike-timing dependent plasticity (STDP) is a biologically constrained unsupervised form of learning that potentiates or depresses synaptic connections based on the precise timing of pre-synaptic and post-synaptic firings. The effects of on-going STDP on the topology of evolving model neural networks were assessed in 50 unique simulations which modeled 2 h of activity. After a period of stabilization, a number of global and local topological features were monitored periodically to quantify on-going changes in network structure. Global topological features included the total number of remaining synapses, average synaptic strengths, and average number of synapses per neuron (degree). Under a range of different input regimes and initial network configurations, each network maintained a robust and highly stable global structure across time. Local topology was monitored by assessing state changes of all three-neuron subgraphs (triads) present in the networks. Overall counts and the range of triad configurations varied little across the simulations; however, a substantial set of individual triads continued to undergo rapid state changes and revealed a dynamic local topology. In addition, specific small-world properties also fluctuated across time. These findings suggest that on-going STDP provides an efficient means of selecting and maintaining a stable yet flexible network organization.

## Introduction

Determining how populations of interacting neurons self-organize in the presence of noisy, rapidly changing stimuli and variable temporal and physical constraints remains a major challenge in neuroscience. In addition to the complex unfolding of developmental programs and environmentally-driven modifications which occur early in life, neural networks continue to evolve throughout the lifespan. This on-going process of self-organization among interacting neurons is the central concern of this report.

One form of self-organization that has been extensively explored is spike-timing dependent plasticity (STDP). As the name implies, STDP is a form of synaptic plasticity where changes in synaptic strength are determined by the precise timing of pre-synaptic and post-synaptic spikes: when the pre-synaptic neuron fires before the post-synaptic neuron, the synapse is potentiated, and when the post-synaptic neuron fires prior to the pre-synaptic neuron, the synapse is depressed. STDP has been observed in a wide-range of neural systems, from mammalian cortical and sub-cortical networks to non-mammalian nervous systems (for a review, see Dan and Poo, 2006 or Froemke et al., 2010).

A number of models have been employed to explain some of the basic properties and consequences of STDP (e.g., Song et al., 2000). One approach used to characterize the potential functionality of STDP has been to explore how it influences the structure (topology) of self-organizing neural networks. The use of graph theoretic measures has revealed a number of adaptive topological features which emerge from STDP-governed networks. For example, Shin and Kim (2006) demonstrated that a model network of excitatory and inhibitory neurons that employed STDP synaptic modification developed small-world properties and power-law degree distributions. Small-world characteristics (i.e., high clustering and short average path lengths; Watts and Strogatz, 1998) have been detected in macaque and cat cortical networks and the human reticular formation. Neuroimaging studies have revealed both small-world properties and power-law degree distributions in human functional brain networks (for a review of graph theoretical analyses of brain networks, see Reijneveld et al., 2007; Bullmore and Sporns, 2009). More recently, Ren et al. (2009) compared the local topological characteristics of the organism *Caenorhibditis elegans* to a biologically inspired model STDP network. They found that the residual network evolved by STDP produced specific three-neuron connectivity patterns (motifs) in significantly greater frequencies than observed in comparable random networks. The profile of significant motif types detected in their model network was qualitatively similar to those observed in the *C. elegans* connectome. These studies suggest that STDP may be an underlying mechanism for the evolution of neuronal topologies observed in some organic neurobiological systems.

While these and other studies have been informative regarding the role of STDP in selecting residual network architectures, they do not address the continual, experience-dependent changes in network architecture which may emerge during on-going STDP. Several recent reports suggest that, in addition to its role in guiding cortical and subcortical structure during development, STDP continues to modify synaptic connectivity in mature neural networks. Yu et al. (2009) demonstrated that neurons in the superior colliculus of adult cats adapted their responses to cross-modal sensory stimuli over short time scales in a manner that was consistent with STDP-governed synaptic modification. The authors suggest that STDP may remain a viable mechanism for rapid structural modification well into adulthood. More recently, Gambino and Holtmaat (2012) showed that receptive fields in mouse barrel cortex could be modified in a timing dependent manner by deflections of surrounding whiskers. These results support a previous study of spike-timing dependent synaptic depression elicited in the primary somatosensory cortex of anesthetized adult rats through the pairing of whisker deflections with spontaneously emitted postsynaptic spikes or spikes generated by current injection (Jacob et al., 2007).

To date, there have been very few studies that address the on-going topological dynamics of neural and brain systems. Robinson et al. (2009) tested the robustness and stability of different model network topologies during dynamic restructuring. Limitations of this study include a restructuring scheme that was not based on a biologically-relevant synaptic modification, and an analysis that was focused on properties of the residual, rather than the evolving, architecture. In another study, Grindrod and Higham (2010) used a functional brain network to demonstrate the effectiveness of new algorithms in characterizing evolving graphs. Although interesting, the network was derived from a short sample of time-series EEG data, and the algorithm assessed only transient functional connectivity over a brief period of time where structural plastic changes were unlikely to take place.

Synaptic changes, including STDP, continue to affect neural organization in living organisms throughout their lifetimes. Whereas other studies have examined the effects of these changes after an initial period of reorganization, the continual evolution of neural network topology shaped by these changes has not been investigated. The aim of the present study was to systematically evaluate the effects of STDP on the on-going dynamic topology of model neural networks. Ten networks were initially randomly connected and then subjected to different types of external input while synapses underwent continual STDP modification. After an initial period, the structural features of the networks were monitored at regular intervals. This novel approach permitted us to detect and track periodic changes in the topology of the networks across time rather than only characterizing the network at a single timepoint. By using small model networks, we were able to simultaneously monitor multiple topological features: changes in the total number of synapses, the average synaptic strengths, and the average number of synapses per neuron were quantified. Changes in the degree of clustering and the average synaptic distance between neurons were also measured to assess changes in the small-world properties of the networks. In addition, changes in local topology and motif profiles were evaluated by monitoring changes in specific three-neuron connectivity patterns across time. Results revealed that with on-going STDP (1) global network structure remained highly stable across time, (2) both stable/persistent and transient/dynamic local features emerged (3) small-world network properties fluctuated, (4) a large and rapid turn-over in local network constituents across time occurred, and (5) qualitatively different forms of network input altered the effects of STDP on topological changes. These effects were robust across a range of variations in STDP parameters and initial network properties.

## Materials and methods

### Model neural networks

The model neural networks used in all simulations consisted of 400 regular-spiking excitatory (RSE) neurons and 100 fast-spiking inhibitory (FSI) interneurons. The neurons of each network were initially connected at random. The in-degrees and out-degrees (i.e., the number of pre- and post-synaptic connections) of each neuron were selected such that there were ~50 pre-synaptic and 50 post-synaptic synapses for each neuron (means = 50, *SD* = 5, normally distributed). Thus, there were ~25,000 synapses (16,000 excitatory-to-excitatory synapses) in each initial network (~10% of full connectivity).

Izhikevich-type neurons were used in all network simulations (Izhikevich, 2004). The neuronal dynamics of both neuron types (RSE and FSI) were modeled by a system of differential equations:

(1) $$\frac{dV}{dt}=0.04V^{2}+5V+140-u+I$$

(2) $$\frac{du}{dt}=a\left(bV-u\right),$$

where ***V*** is the neuronal membrane voltage (in millivolts, mV), ***u*** is a membrane relaxation variable, and ***I*** is the total input to the neuron (in mV). An action potential (spike) occurred when ***V*** ≥ 30 mV, after which the voltage and relaxation variables were reset according to the equation:

(3) V≥30:{V←c;u←(u+d)}

Equations 1, 2, and 3, together with a range of values for parameters ***a***, ***b***, ***c***, and ***d***, allow a great variety of neuronal types and dynamics to be modeled. Parameters ***a*** and ***b*** are rate variables that determine how quickly each neuron recovers from a depolarizing event, while parameter ***c*** is the resting membrane potential, and parameter ***d*** is an additional resetting variable following an action potential. Based on previous work (Izhikevich, 2004), the parameters for the RSE neurons were set to: ***a*** = 0.02, ***b*** = 0.2, ***c*** = −65 mV, and ***d*** = 8. For FSI neurons, the parameters were set to: ***a*** = 0.1, ***b*** = 0.2, ***c*** = −65 mV and ***d*** = 2. Note that parameters affecting the relaxation variable, ***u***, are modified for FSI neurons to shorten post-firing recovery which gives these neurons their fast-spiking behavior. The values of ***u*** and ***V*** were approximated using a fourth-order Runge–Kutta numerical method to evaluate Equations 1, 2, and 3 (*h* = 0.5 timesteps for Equation 1, and one timestep for Equations 2 and 3). The simulation timestep was selected to approximate 1 ms of real time.

The excitatory post-synaptic weights were initially uniformly distributed between 0 and 8 mV (RSE → RSE synapses and RSE → FSI synapses), while initial inhibitory post-synaptic weights were distributed between −8 and 0 mV (FSI → RSE synapses and FSI → FSI synapses).

### Network input

Each network received five different types of external input (input regimes) in separate simulations. External stimulation varied in degree of regularity (periodicity) and synchrony and was qualitatively similar to the distinct types of spiking network dynamics outlined by Brunel (2000).

#### Regular synchronous input (RS)

Under the regular, synchronous input regime, a randomly selected subset of all neurons (mean = 100 neurons, *SD* = 1, normally distributed) received 16 mV input simultaneously every 20 timesteps (20 ms intervals, 50 Hz input rate). The subset of neurons receiving input changed on every input cycle (i.e., every 20 timesteps).

#### Regular asynchronous input (RA)

The input parameters of the regular but asynchronous regime were the same as RS input except that the input timing to each neuron was jittered around a mean of 20 ms with a 6 ms standard deviation.

#### Irregular synchronous input (IS)

The third input regime simulated irregular, synchronous input. Similar to RS input, a randomly selected subset of approximately 100 neurons received simultaneous input; however, input was delivered at a Poissonian distributed rate with a mean of 50 Hz (i.e., non-periodic input).

#### Irregular 50 Hz input (IA50) and irregular 12 Hz input (IA12)

In the final two input regimes, input was delivered irregularly and asynchronously such that every neuron in the network received 16 mV input independently at a Poissonian distributed mean rate of either 50 or 12 Hz.

In addition to external input, every neuron in the network received a constant small subthreshold input at each timestep throughout the simulations. The value of this noisy input was randomly selected at each timestep from a Gaussian distribution with mean of 1.3 mV and standard deviation of 0.5 mV.

In addition to external input and subthreshold input, neurons in the network received synaptic input from their pre-synaptic neurons whenever the pre-synaptic neuron fired. The input to the post-synaptic neuron occurred one timestep after the pre-synaptic neuron fired (1 ms delay). The magnitude of synaptic input was equal to the weight of the synapse from the pre-synaptic neuron. The value of ***I*** in Equation 1 was taken as the sum of these three input terms for each neuron at every timestep.

### Spike-timing dependent plasticity

The weights of the RSE → RSE synapses in the network were modified for the duration of the simulation according to an additive STDP learning rule. The STDP rule was implemented such that a synapse was potentiated if a pre-synaptic neuron fired before a post-synaptic neuron according to the equation:

(4) $$\Delta \omega_{t}=\Delta \omega_{t-1}+A_{+}e^{-\left(tpost-tpre\right)/\tau }$$

If the post-synaptic neuron fired before the pre-synaptic neuron, the synapse was depressed according to the equation:

(5) $$\Delta \omega_{t}=\Delta \omega_{t-1}+A_{-}e^{-\left(tpost-tpre\right)/\tau }$$

In Equations 4 and 5, Δ ω_***t***_ is the additive (±) change in synaptic weight, ***t*post** and ***t*pre** are the post-synaptic and pre-synaptic firing times, respectively, Δ ω_***t*−1**_ is the value of Δ ω from the preceding timestep. The values of τ, ***A***_+_, and ***A***_−_ were selected based on the empirically derived STDP model outlined in Song et al. (2000). The width of the STDP time window was determined by the time constant τ = 20 ms. The values of ***A***_+_ and ***A***_−_ determine the maximum or minimum weight change for a given synaptic event (learning rate). The value of ***A***_+_ was set to 0.044 which is 0.55% of the maximal weight attainable by any synapse (8 mV). The value of ***A***_−_ was set to −0.0462 to produce an asymmetry (bias toward synaptic depression) in the STDP rule. This bias was introduced so that uncorrelated pre-synaptic and post-synaptic firings result in an overall weakening of the synapse.

The STDP rule was applied at all excitatory-to-excitatory synapses (RSE → RSE). Inhibitory synaptic strengths remained constant. Studies show that STDP at inhibitory synapses likely follows different STDP rules (e.g., Haas et al., 2006). STDP was not applied at these synapses to avoid potentially misleading effects and to increase computational tractability. Δω was initially set to zero, and Equations 4 and 5 were evaluated during each timestep of the simulation. The synapse was updated once every 1000 timesteps (1 s intervals) by adding the current value of Δω at that time point to the current weight. Synaptic weights were bounded so that when the sum of the synaptic weight and Δω was >8 mV or <0 mV, the weight was set to 8 mV or 0 mV, respectively.

To simulate on-going STDP and permit re-potentiation of zero-weight synapses, synapses with 0 mV weights were not removed from the network. Instead, the value of Δω continued to be modified by Equations 4 and 5 at each timestep and added to the weight after every 1000 timesteps.

### Simulations and analysis

The dynamic effects of STDP on network topology were examined in 10 separate model networks. Each network received each of the five external input regimes (RS, RA, IS, IA50, and IA12) in separate simulations resulting in 50 simulations total. Each simulation consisted of 7.2 million timesteps, or 2 simulated hours of activity. Measures of global and local topological features were sampled once every 60,000 timesteps (1 min intervals) during the last half of each simulation (The analysis interval; timesteps 3,600,001–7,200,000).

#### Global measures

Global measures included the total number of excitatory-to-excitatory synapses in the network, the average weight of these synapses, and the average synaptic degree (the total pre-synaptic and post-synaptic excitatory-to-excitatory connections) per neuron at each sampling point.

In order to assess the stability of each of these measures across time, the coefficient of variation was also calculated for each measure, where the coefficient equals the standard deviation of the measure across the analysis interval divided by its mean. A smaller coefficient indicates more stability (i.e., less variation) across time. The coefficient of variation is a dimensionless quantity that permits direct comparisons of temporal stability between networks/simulations in circumstances where comparisons of means would be uninformative.

#### Small-world features

The potential small-world characteristics of the networks were also evaluated by calculating the average clustering coefficients and path lengths across all excitatory neurons at each sampling point in the analysis interval. The clustering coefficient is a measure that reflects the likelihood that two connected neurons (neighbors) would share a common neighbor. The clustering coefficient was determined in a manner which took into account both the strengths and directions of the synapses involved in each cluster and was based on a method presented in Fagiolo (2007). The clustering coefficient of each neuron was evaluated by the equation

(6) $$C_{i}=\frac{2}{k_{i}\left(k_{i}-1\right)-2\overset{↔}{k_{i}}}\sum_{j,m}{\left(w_{ij}w_{im}w_{jm}\right)}^{1/3},$$

where ***C***_*i*_ is the clustering coefficient of neuron ***i***, and ***k***_*i*_ is the total number of in-degrees and out-degrees of that neuron. $\overset{↔}{k_{i}}$ is the sum of bi-directional synapses of neuron ***i*** and its neighbors (i.e., where neuron ***i*** is both a pre-synaptic and post-synaptic neuron with its neighbor). ***w***_*ij*_, ***w***_*im*_, and ***w***_*jm*_ are the synaptic weights between neuron ***i*** and its neighbor ***j***, neuron ***i*** and its neighbor ***m***, and the weight between the two neighbors, respectively. Note that Equation 6 only describes clusters that are purely directional (by subtracting out the bi-directional synaptic term, $\overset{↔}{k_{i}}$ from the denominator) and scales these clusters by the geometric mean of their synapses.

Path lengths were also determined in a manner that accounted for directionality and synaptic strength by employing Dijkstra's Algorithm (Dijkstra, 1959). This algorithm assumes that the shortest distance between neuron ***i*** and neuron ***j*** is the directed distance between them containing the fewest synapses of the lowest magnitude. In the networks considered here, greater synaptic strengths should decrease distance (i.e., shorten paths). Since Dijkstra's Algorithm “punishes” synapses of greater magnitude and rewards synapses of lower magnitude, the inverses of synaptic weights were used to assess path length.

Clustering coefficients and path lengths were averaged across all neurons at each sampling timepoint. The averages from each simulation were compared to the values from the initial randomly connected networks. Higher clustering values and shorter path lengths than in the initial networks indicated an improved small-world topology. Functions included in the Brain Connectivity Toolbox (Rubinov and Sporns, 2010) were modified and employed to calculate these measures.

#### Triads

**Figure 1:**
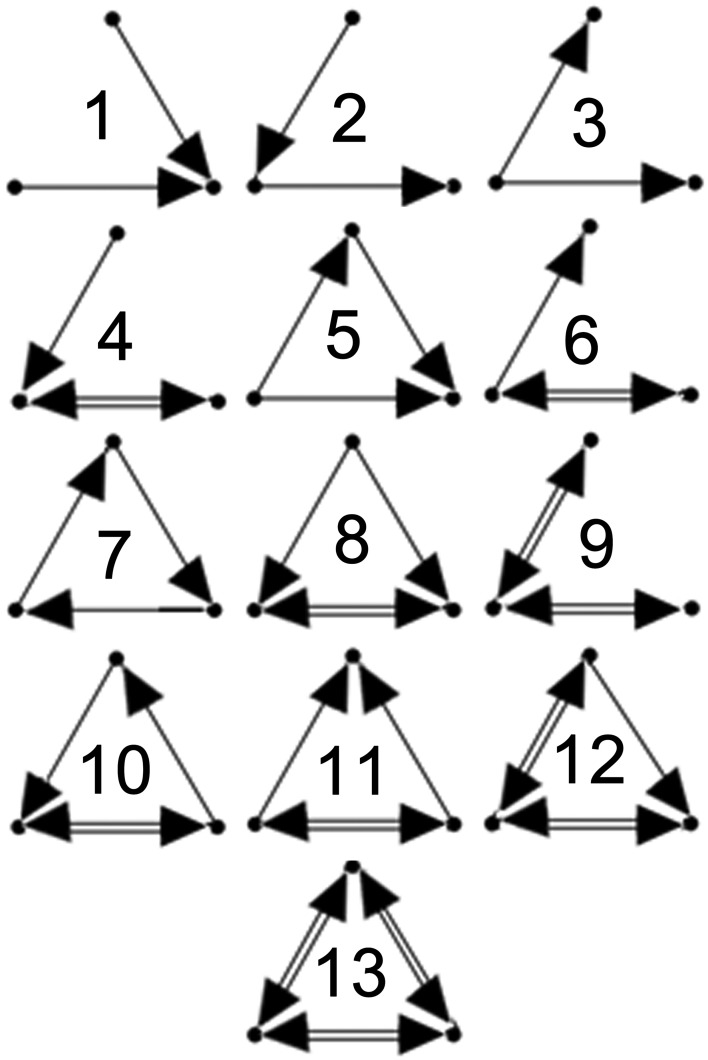
**Triad types.** Configurations of the 13 possible unique 3-neuron subgraphs (triad types). Numbering scheme taken from Sporns and Kotter (2004).

In order to quantify the local topological dynamics of each network, all three-neuron connected subgraphs (triads) present in each network at initialization were identified and changes in their synaptic connectivites were monitored during the analysis interval. There are 13 possible unique connectivity patterns in three-neuron subgraphs (Figure 1). The distributions of these 13 triad types in each network and their synaptic weights were assessed. Since only RSE → RSE synapses were subjected to the STDP learning rule, only triads comprised exclusively of excitatory neurons were considered in the analysis. Although on-going STDP permits the depression and repotentiation of existing synaptic connections, new synapses cannot be formed. Therefore, all of the individual triads identified in each of the 10 initial networks may undergo state changes, but new triads cannot appear.

#### Triad intensity and coherence

To determine the strength and stability of each triad, the synaptic intensities and coherences of the triad were measured during the analysis interval according to a method outlined by Onnela et al. (2005). The synaptic intensity of a triad is equivalent to the geometric mean of its synapses:

(7) $$I(s)={\left(\prod_{\left(i,j\right)\in N_{s}}w_{ij}\right)}^{1/N_{s}},$$

where ***I(s)*** is the intensity of triad ***s***, ***N*_*s*_** represents the number of synapses in ***s***, and ***w***_*ij*_ refers to the synaptic strength of neuron ***i*** onto neuron ***j***. In the present scheme, intensities could range from 0 mV (non-connected) to 8 mV (when every synapse is at maximal weight). In addition, triad coherence is defined as the intensity of the triad (geometric mean of its synapses) divided by the arithmetic mean of its synapses:

(8) $$H(s)=\frac{I(s)}{\sum_{\left(i,j\right)\;\in N_{s}}w_{ij}},$$

where ***H(s)*** is the coherence of triad ***s***. Because the geometric mean (intensity) of the synapses is always less than the arithmetic mean except when all of the synapses are of equal weight, coherence measures biases that occur when some synapses forming the triad are stronger than others. The coherence of a triad is bounded between 0 and 1, and values approaching 1 occur when the synapses of the triad have nearly equal weights (i.e., when the geometric mean approaches the arithmetic mean). Thus, coherence is a measure of triad synaptic stability.

#### Triad turn-over

As a final measure of triad dynamics, the total number of individual triads that re-emerged at a sampling point (triads gained), the total number that disappeared at that sampling point (triads lost), and the net change in the total number of triads (triads gained–triads lost) from one sampling point to the next were calculated.

#### Comparing input regimes

For each of the topological measures, the effects of external input regime were assessed by performing a univariate analysis-of-variance (ANOVA) test on the measure where external input type was the fixed factor. When this test yielded a significance value of *p* = 0.05, separate *t*-tests were performed which contrasted the effects of synchronous vs. asynchronous input (RS and IS vs. RA, IA50, and IA 12) and regular vs. irregular input (RS and RA vs. IS, IA50, and IA 12). These tests were Bonferroni corrected.

## Results

### Input dependent steady-state network activity emerges during on-going STDP

**Figure 2:**
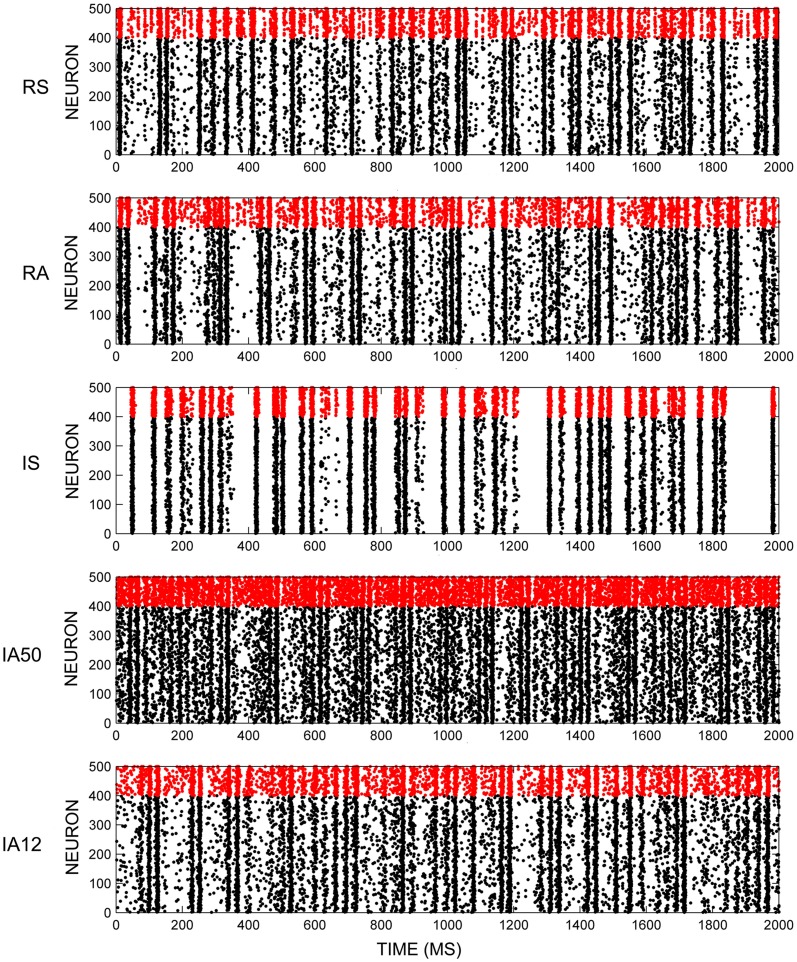
**Network firing activity.** Exemplar rastergrams of 2 s of network firing activity in response to different input regimes. All examples are from the same initial network. Red data points are FSI firings, black data points are RSE firings.

Early activity in each initial randomly configured network displayed a pattern of “synfire explosions,” where brief periods of inactivity were separated by periods when all of the neurons fired synchronously. This activity resulted in the initial loss of a large percentage of weak synapses. However, a pattern of steady-state synchronous firing activity emerged shortly after the beginning of each simulation (~2 min). Figure 2 shows exemplar network activity for each of the external input regimes. During this steady-state, fluctuations in the average neuronal firing rates across time were marginal. Rates were determined by external stimulus type where irregular input patterns (IS, IA50, IA12) significantly increased firings of excitatory neurons compared to regular input patterns [*t*_(48)_ = 3.52, *p* = 0.001]. However, firing rates for RSE neurons from all simulations remained between 12 and 16 Hz. FSI neurons fired at almost twice that rate (overall mean = 29.68 Hz) owing to parameter differences in Equations 2 and 3.

### A stable global network structure emerges from on-going STDP

**Figure 3:**
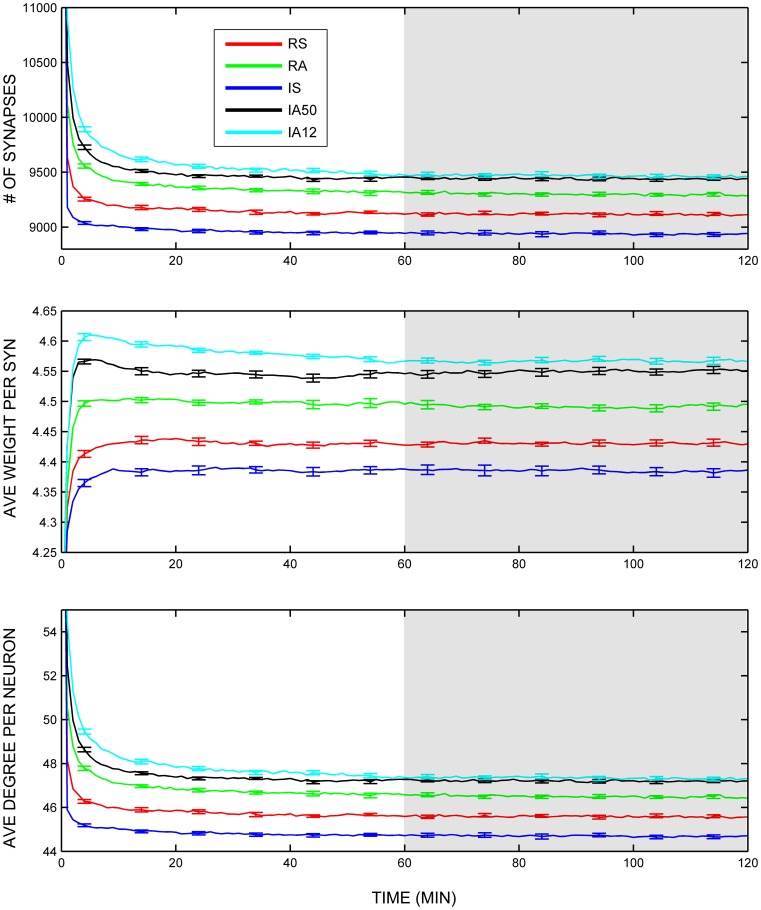
**Global variables.** Trajectories of total number of excitatory synapses **(top)**, average synaptic weight **(middle)**, and average total neuronal degrees **(bottom)** across the entire simulation duration. Colored lines represent responses to separate external input regimes averaged across 10 simulations. Error bars represent S.E.M. across each of the 10 simulations at that timepoint. Shaded area represents analysis interval. Note that each input regime started with that same initial number of synapses (mean = 15,991.80), weights (mean = 4.00 Hz), and degrees (mean = 79.96) although they exceed the range of the graphs.

Global topological features including the total number of synapses, their average synaptic weight, and average neuronal degree (number of excitatory synapses per neuron) were collected throughout the simulations and evaluated across the analysis interval (during the last half of the simulation). Figure 3 displays the trajectories of these measures across the simulations. Although the range of values was narrow across the different types of external input, all three measures were significantly influenced by synchronous input regimes. The numbers of remaining synapses were significantly fewer during synchronous input regimes (RS, IS) compared to asynchronous input regimes [RA, IA50, IA12; *t*_(48)_ = 13.42, *p* < 0.001; Figure 3, top]. Likewise, the average weight per synapse was also significantly reduced during synchronous input compared to other input types [*t*_(48)_ = 13.32, *p* < 0.001; Figure 3, middle]. The average neuronal synaptic degree for excitatory neurons showed a similar pattern [*t*_(48)_ = 13.87, *p* < 0.001; Figure 3, bottom].

**Figure 4:**
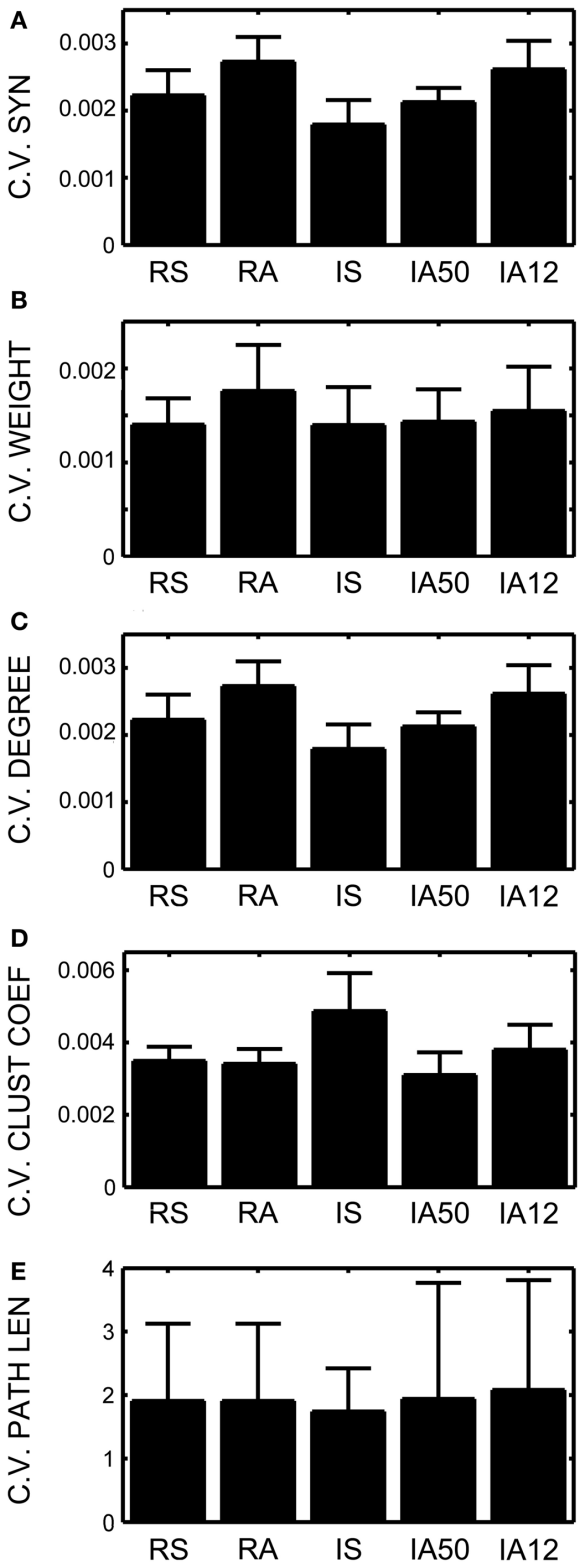
**Coefficients of variation across time.** Coefficients of variation (the standard deviation across time divided by the mean) for **(A)** Total number of excitatory synapses, **(B)** Average synaptic weight, **(C)** Average total neuronal degrees, **(D)** Average weighted and directed clustering coefficient, and **(E)** The average weighted and directed path length. Error bars represent SD's across the 10 simulations for each input regime.

To better characterize how these global measures were changing during the simulations, the coefficient of variation was assessed for each input regime. Lower coefficients signal less variability across time, or greater stability. In all cases, the coefficients remained extremely low suggesting a very high degree of stability (Figures 4A–C). Nevertheless, external input type again exerted a significant influence. Synchrony produced significantly less change in the numbers of synapses, and in the number of synaptic degrees [*t*_(48)_ = 3.93, *t*_(48)_ = 3.92, *p* < 0.001 both cases]. Interestingly, coefficients of variation for both measures were practically identical despite obvious differences in the means and standard deviations.

Small-world topology was also evaluated across the analysis interval. The overall average clustering coefficient for RSE → RSE neurons across the analysis window for all simulations was 0.435 (*SD* = 0.006). In every simulation, the clustering coefficient was greater during the analysis interval than at initialization (initial network mean = 0.3368, *SD* = 0.0022), suggesting increased clustering due to on-going STDP and external input. Clustering coefficients varied little across the analysis interval (i.e., low coefficient of variation, Figure 4D). The degree of clustering was significantly affected by external input type as was the coefficient of variation such that synchronous input decreased clustering [*t*_(48)_ = 8.67, *p* = 0.003] but also increased its variability across time [*t*_(48)_ = 3.10, *p* = 0.009].

In contrast, the average shortest path length increased almost an order of magnitude over its value at initialization (path length mean at initialization = 0.3537, *SD*< 0.001; mean of overall path length during analysis interval = 3.196, *SD* = 2.03). Average path length fluctuated greatly during the simulations, as is apparent in the high coefficients of variation for all input regimes (Figure 4E). Unlike clustering, path length was not differentially affected by external input regime.

### Three-neuron subgraphs (triads) reveal local topological dynamics

An average of 1,002,498 unique triads were present in the 10 original random networks, (*SD* = 7216.74). In general, the number of these triads that remained in each network (i.e., that were detected at least once during the analysis interval) was a small fraction of those present at initialization. Table 1 shows the total number and percentages of triads detected. The percentage of total triads that remained varied significantly according to input type, where synchronous input regimes resulted in significantly smaller percentages of remaining triads [*t*_(48)_ = 13.47, *p* < 0.001].

**Table 1:** **Core and dynamic triad characteristics by input regime**.

	**Input regime**				
	**RS**	**RA**	**IS**	**IA50**	**IA12**
Total no of triads	516343.6 (8650.1)^*^	556875.1 (9821.11)	465299.3 (9944.84)^*^	566319.7 (10173.93)	569135.4 (9473.55)
%Remaining	51.51 (0.73)^*^	55.55 (0.85)	46.41 (0.89)^*^	56.49 (0.9)	56.77 (0.81)
**CORE TRIADS**					
%of TTL	54.52 (2.34)^*^	49.75 (1.95)	61.39 (2.68)^*^	48.78 (1.95)	50.08 (2.2)
Intensity	7.99 (0.047, <0.001)^*^	7.99 (0.064, <0.001)	7.99 (0.044, <0.001)^*^	7.98 (0.061, <0.001)	7.98 (0.058, <0.001)
Coherence	1 (0.003, <0.001)^*^	1 (0.004, <0.001)^*^	1 (0.002, <0.001)^*^	0.999 (0.003, <0.001)	1 (0.003, <0.001)
**DYNAMIC TRIADS**					
%of TTL	45.48 (2.34)	50.25 (1.95)^*^	38.61 (2.68)	51.22 (1.95)^*^	49.92 (2.2)^*^
Intensity	4.77 (2.823, 0.19)^*^	4.67 (2.802, 0.09)	5.39 (2.798, 0.19)^*^	4.77 (2.828, 0.14)	4.7 (2.81, 0.17)
Coherence	0.7 (0.303, 0.02)^*^	0.69 (0.298, 0.01)	0.76 (0.287, 0.02)^*^	0.71 (0.281, 0.01)	0.69 (0.294, 0.02)

Values represent means across simulations. Values in parentheses represent SD's across triads (where appropriate) and/or networks, respectively.

* Indicates significant difference in measure due to input regime.

In all of the simulations, the set of remaining triads was divided between core triads, which showed no change in their connectivity pattern throughout the analysis interval, and dynamic triads, which disappeared, re-emerged, or changed triad type at least once during analysis. Table 1 displays demographics for the core and dynamic triads for each input regime. Again, the percentages of the core triads and dynamic triads present in each network depended upon the external input type each received such that synchronous input significantly increased the proportion of core triads relative to asynchronous input [*t*_(48)_ = 9.31, *p* < 0.001].

### Core triads consist of strong and stable synapses

**Figure 5:**
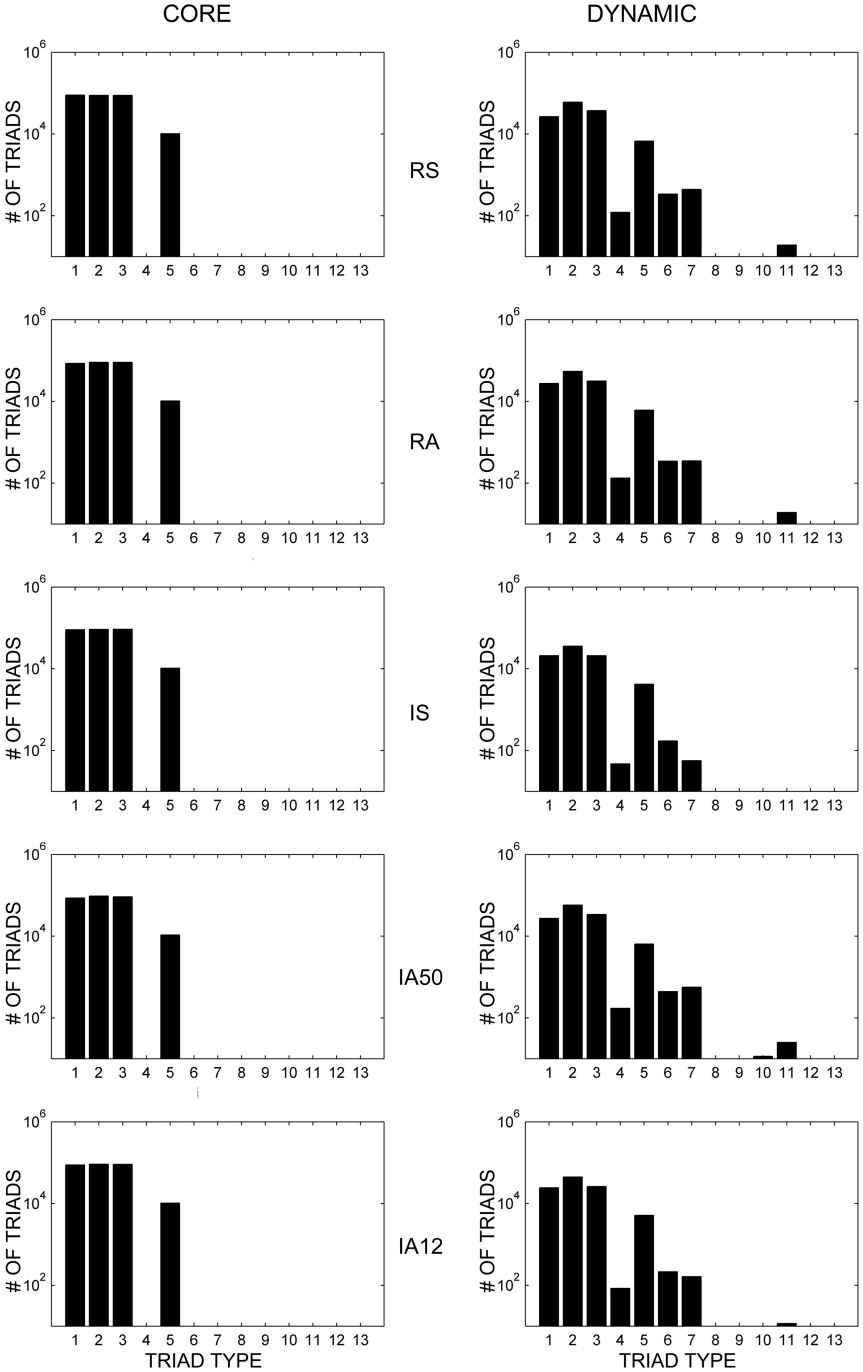
**Core and dynamic triad type distributions.** Triad type distributions for core **(left)** and dynamic **(right)** triads in response to each external input regime averaged across 10 simulations. Dynamic triad distributions are averaged across the analysis interval for each network and then across simulations. Note that triad counts (ordinate axes) are presented on a logarithmic (base 10) scale. Note that due to logarithmic scaling, triad types with very small averages are not visible.

Figure 5 (left) displays the distributions of the 13 possible triad types (see Figure 1) of core triads averaged across the different input regimes. Two-synapse core triad types (types 1, 2, and 3) occurred in every simulation, as did core triads of type 5. Type 7 core triads were observed in several of the simulations involving asynchronous input (IS, IA50, IA12). Note that because the numbers of core triads of this type were relatively small, this type is not visible in the figure due to the broad logarithmic scale of the distribution. No other triad types consistently retained their initial configuration during the simulations.

The strength and stability of core triads were determined by assessing their triad intensities (geometric means) and coherences. Core triad intensities from all simulations approached maximal values (overall mean = 7.99 mV) as did core triad coherences in all simulations (overall mean > 0.999). The high values of core intensities and coherences suggest that these triads consisted of strong synapses of nearly equal weight. The values of intensities and coherences from all of the simulations were very similar (overall *SD* of intensity = 0.005; *SD* of coherence < 0.001). Nevertheless, a significant effect of external input type was detected. Regular regimes reduced the coherences of core triads [*t*_(48)_ = 2.46, *p* = 0.017] while synchronous regimes slightly increased both core intensities [*t*_(48)_ = 14.26, *p* < 0.001] and core coherences [*t*_(48)_ = 3.33, *p* = 0.002] relative to other input types (Table 1).

### Dynamic triads reveal an active local topology

Dynamic triads are those triads which were detected during analysis but which changed connectivity pattern at least once during the analysis interval. Whereas synchrony significantly increased the proportion of core triads observed, asynchronous input increased the proportion of dynamic triads.

Figure 5 (right) displays the distributions of the different types of dynamic triads averaged across time and simulations. Similar to core triad distributions, dynamic triad types consisting of two synapses (types 1, 2, and 3) and triad type 5 occurred most frequently; however, dynamic triads were more diverse and every triad type was detected at least once during the simulations (although those that occurred rarely are not visible in the figure). Because dynamic triads can change type, distributions represent the average number of types across the analysis interval rather than sums of individual triads. Despite the dynamic range of these triads, the distribution of triad types remained highly stable across time (overall coefficient of variation across all triad types = 0.865).

**Figure 6:**
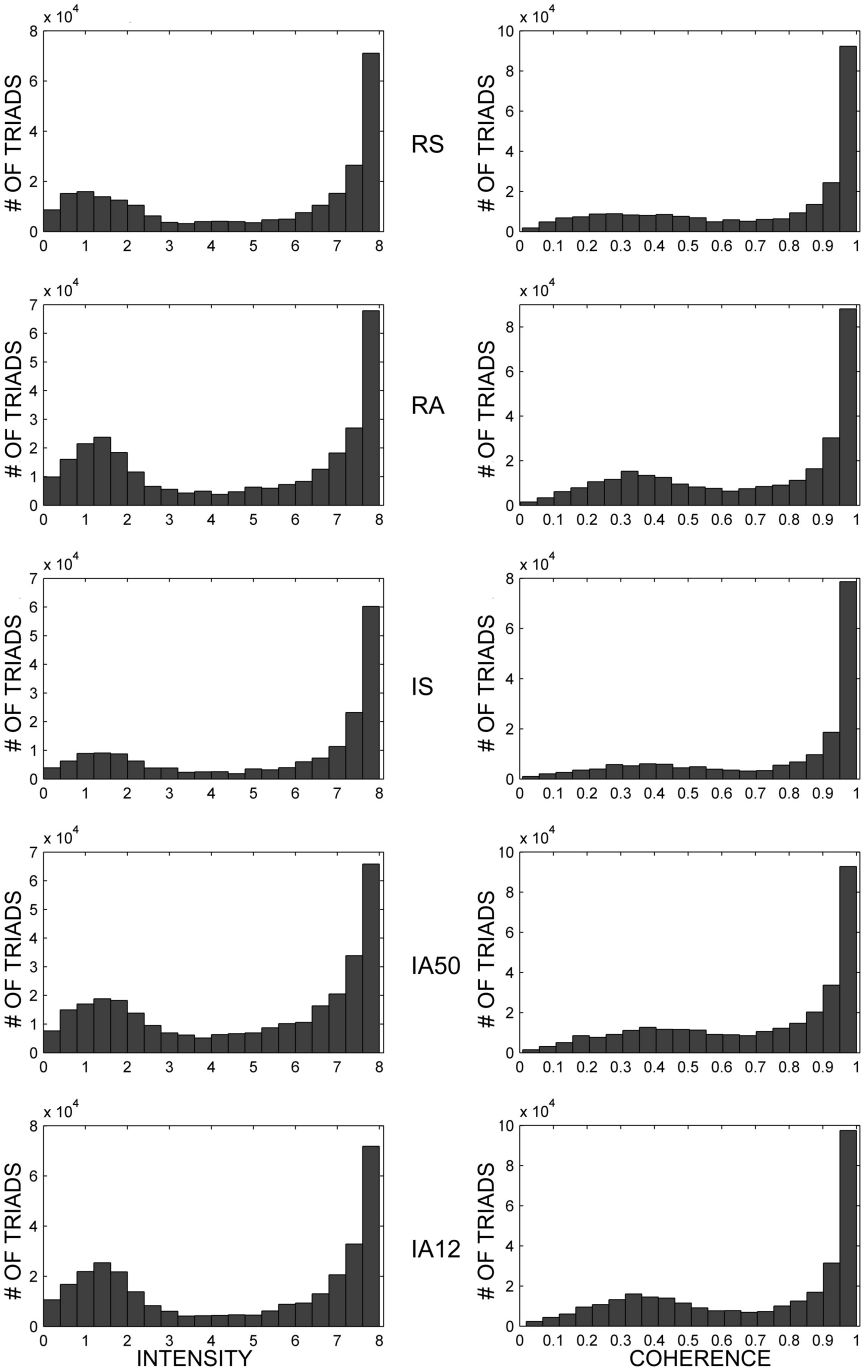
**Dynamic triad intensities and coherences.** Exemplar dynamic triad intensity **(left)** and coherence **(right)** distributions in response to separate external input regimes. All examples are from the same initial network. Note that triad counts (ordinate axes) are not on the same scale for all input regimes, and all intermediate ordinate values are scaled by a factor of 10^−4^.

Example distributions of the average intensities and coherences of dynamic triad types for each external input regime are shown in Figure 6, and their values are presented in Table 1. Intensities and coherences from all simulations displayed a bimodal and skewed distribution. The bimodal and negatively skewed distributions suggests that a large proportion of these triads possessed relatively strong, stable synapses despite on-going state changes while a smaller proportion were more unstable. Alternatively, this distribution pattern may reflect high frequencies of individual triads which possessed both strong-stable, and weak-unstable states. The intensities and coherences of synapses forming dynamic triads varied according to input type. Similar to core triads, regular input regimes (RS, RA) significantly reduced both the intensity [*t*_(48)_ = 2.72, *p* = 0.009] and coherence [*t*_(48)_ = 3.18, *p* = 0.003] of dynamic triads. Synchronous input significantly increased both measures [intensity, *t*_(48)_ = 4.98; coherence, *t*_(48)_ = 4.11; *p* < 0.001 both].

### Individual triads disappear and re-emerge at rapid rates

**Figure 7:**
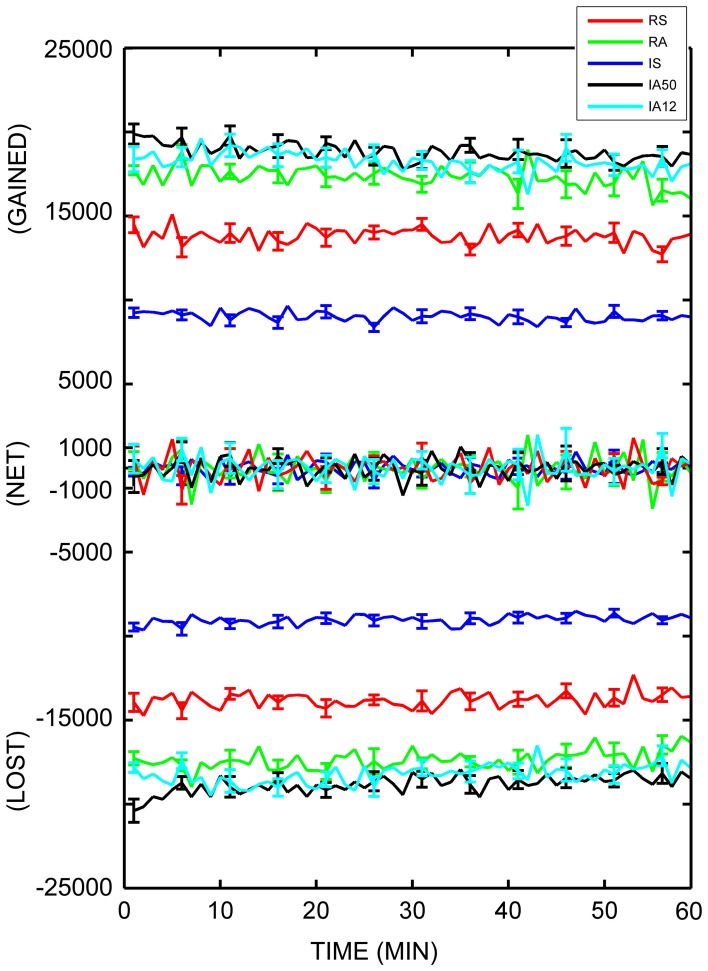
**Dynamic triad trajectories.** Average number of dynamic triads gained, lost, and net triad number differences for each minute in the analysis interval. Each minute (abscissa) represents the difference between that minute and the following minute. The number of individual triads (ordinate axis) is separated into “gained,” “net,” and “lost” for clarity. Colored lines represent responses to separate external input regimes averaged across 10 simulations. Error bars represent S.E.M. across each of the 10 simulations at that timepoint.

To fully assess the dynamics of the local topology, the numbers of individual dynamic triads which disappeared and re-emerged across each sampling interval were calculated. The average number of individual triads which were lost from one sampling point to the next was 15,459.25 (*SD* = 3765.55), while the number that were gained (re-emerging as either as the same type or as a different type) was 15,444.37 (*SD* = 3762.79). Table 2 and Figure 7 display the average number of individual triads that were gained and lost at each sampling point, as well as net changes in total number of triads. As is evident from the figure and table, nearly as many triads were gained at each timepoint as were lost for each input regime, keeping the overall number of triads across time negligible. For all simulations, the average change in the total number of triads from one sampling point to the next (net change) was ±1676.47 triads (*SD* = ± 378.78). To better quantify the disparity between the numbers of individual triads which disappeared and re-emerged and the overall changes in triad counts, the ratio of the number of triads which re-appeared at each sampling point to the net change in triad counts was calculated. The overall gained-to-net ratio was 9.26 (*SD* = 1.54) across all simulations, almost a tenfold increase. This implies that the total number of triads remained nearly constant while the composition of participating triads was changing at nearly 10 times the rate. External stimulus type influenced triad losses, gains, and net changes. In every case, synchronous input significantly reduced these values [*t*_(48)_ = 13.09, 13.06, and 6.79 respectively; *p* < 0.001 for all cases].

**Table 2:** **Triad turn-over by input regime**.

	**Input regime**				
	**RS**	**RA**	**IS**	**IA50**	**IA12**
Net change	1623.23^*^ (1249.73)	1953.81 (1467.43)	1087.04^*^ (806.91)	1716.94 (1234.55)	2001.32 (1511.56)
Triads gained	13802.95^*^ (1373.46)	17340.58 (1666.98)	9025.7^*^ (902.58)	18815.17 (1488.23)	18237.44 (1668.52)
Triads lost	-13811.87^*^ (1283.37)	-17376.07 (1593.05)	-9035.94^*^ (880.07)	-18830.03 (1447.61)	-18242.36 (1588.17)
Gained-to-net ratio	8.71^*^ (1.1)	8.91 (1.15)	8.36^*^ (1.12)	11.11 (1.21)	9.23 (1.12)

Values represent means across simulations. Values in parentheses represent SD's across time.

* Indicates significant difference in measure due to input regime.

### The final networks possess significant motif types

Different types of small connected subgraphs present in complex networks are frequently referred to as “motifs.” Milo et al. (2002) defined motifs as subgraphs that occur in a network of interest at significantly greater frequencies than observed in equivalent, randomly connected networks of the same size. The significance of occurrences of each of the 13 possible different triad types was tested in the final organizations of the networks investigated (i.e., at the final timestep of each simulation). Thus, triads of significant occurrence were motifs. Final networks were compared to randomly connected networks where the random networks were generated by switching synapses between neurons in the final network while preserving the same number of incoming, outgoing, and mutual synapses of each neuron (mfinder version 1.2, http://www.weizmann.ac.il/mcb/UriAlon). Between 50,000 and 100,000 switches were performed for each graph and 100 random graphs were generated for comparison. Details of the edge switching algorithm can be found in Milo et al. (2003).

For each of the 13 triad types, the number of occurrences of the type in the final network was compared to the number of occurrences of the same type in 100 random networks by calculating a Z-score test statistic as follows:

(9) $$Z=\frac{F_{final}-F_{random}}{\sigma_{random}},$$

where ***F*_final_** is the number of occurrences of the type in the final network and **F_random_** and **σ_random_** are the mean and standard deviation of the occurrences in the 100 random networks. If the Z-score was greater than 1.96 or less than −1.96, the type was considered to have occurred at significantly greater or fewer numbers than in the distribution of random networks (at the 95% confidence level), and therefore represented a significant motif in the final network.

The numbers of significant triad types (motifs) found in the final networks for each input regime were similar to those detected previously in model and biological neural networks (Ren et al., 2009). In every simulation, triad types 2 and 5 were found to be significant motifs, occurring more frequently than in random networks. Triad type 8 also occurred significantly more frequently across all input regimes. Triad types 1, 3, and 7 occurred significantly less frequently in all simulations. Other motif types revealed more complex patterns. Type 4 occurred significantly more after one simulation of the RS condition and significantly less after irregular input (IS, IA50, IA12) following several simulations. Type 6 occurred significantly more following one IS simulation and one IA50 simulation and less in one simulation following RA input. Type 10 occurred significantly more frequently following one of the IA12 input simulations, but occurred significantly less in several simulations following RS and IA50 input. Finally, type 11 occurred significantly more in one simulation following an RA input simulation and significantly less in one simulation involving IA50 input.

### Variations in STDP learning parameters and network configurations have mixed effects on stability and dynamics

Several parameters of the STDP learning rule (Equations 4 and 5) were modified to assess the robustness and generalizability of the learning rule to effects upon network stability and dynamics:

#### Reduced STDP rate condition

In the first modification, the values of ***A***_+_ and ***A***_−_ (the degree of change in synaptic strength per plasticity event) were reduced to one tenth of their original values (0.0044 and –0.0046, respectively).

#### Reduced STDP window condition

In the second modification, the STDP time constant, τ, was narrowed from 20 to 10 ms.

#### Symmetric STDP condition

In this modification the asymmetry of the learning rule was removed such that ***A***_+_ = ***A***_−_ = 0.044 (i.e., the bias toward synaptic depression was eliminated).

In addition to modifications in the STDP learning rule, several more modifications were made to the initial network parameters:

#### Reduced synaptic weight condition

In the first condition, the permissible range of synaptic weights was reduced from −8 to 8 mV to −4 to 4 mV while all other parameters remained the same.

#### Asymmetric weight condition

In the second condition, an asymmetry was introduced in the excitatory-to-inhibitory synaptic weight ratio such that the range of excitatory synaptic weights remained from 0 to 8 mV while the inhibitory range was increased from 0 to −8 mV to 0 to −9.6 mV.

#### Sparse connectivity condition

In the third condition, the number of initial synapses was evenly decreased from 25,000 to 12,500 (from 10% of full connectivity to 5% of full connectivity).

#### Stationary input condition

In the final modification, a subset of 100 neurons was randomly selected prior to the simulations, and only these neurons received external input. This condition contrasted with the original simulations where the subset of neurons receiving input changed during every input event.

One simulation of each of these conditions was conducted using each of the five external input regimes, resulting in 35 separate simulations. The same initial network was used for each simulation and was selected from the initial networks employed in the original simulations. During the parameter modified simulations, all other parameters remained the same as in the original simulations.

Parameter modified simulations were compared to the original simulations on several measures of stability and dynamics: (1) the coefficient of variation across time of the number of synapses remaining, (2) the coefficient of variation of the average synaptic weight of these synapses, (3) the coefficient of variation of the average neuronal degree, (4) the percentages of core and dynamic triads remaining during the analysis interval, and (5) the gained-to-net ratios of triad turn-over. All of these measures are dimensionless and are thus suitable for comparisons with unmodified networks where quantitative differences occur. Further, they capture qualities of topological change that direct comparisons of means may not.

To assess the magnitude of the effects of these variations upon global and local network stability, direct comparisons were made to the original, unmodified simulations by performing significance tests. *T*-tests were used as the test statistic where ***t*** was calculated as follows:

(10) $$t=\frac{V_{modified}-{\bar{V}}_{original}}{\sigma_{original}/\sqrt{n}}$$

***V*_modified_** is the value of the measure resulting from the modified simulation and ${\bar{V}}_{original}$ and **σ_original_** are the mean and standard deviation of the values from the original simulations, respectively, and ***n*** is the sample size (10 in all cases). T-scores greater than 3.25 or less than −3.25 were considered to be indications of a significant deviation in the measure from values obtained in the original simulations (at the 99% confidence interval). Comparisons were only made between simulations employing the same type of external input (e.g., STDP modified networks receiving RS type stimulation were only compared to the 10 original simulations where RS stimulation was used).

Tables 3, 4 report values of topological features obtained from each parameter variation condition.

**Table 3:** **Global stability and triad dynamics for all parameter variations**.

**Condition**	**Global stability**				**Triad dynamics**			
	**Input**	**C.V. no. of syns**	**C.V. weight**	**C.V. degree**	**Net change**	**Gained**	**Lost**	**Gain/net**
**STDP PARAMETER VARIATIONS**								
Reduced rate	RS	0.0025	0.0005	0.0025	1741.53	13425.65	13406.05	7.7091
Reduced window	RS	0.0015	0.0011	0.0015	1012.09	8032.56	8005.86	7.9366
Symmetric STDP	RS	0.0024	0.0018	0.0024	1493.85	10923.51	10876	7.3123
Reduced rate	RA	0.0032^*^	0.0004^**^	0.0032	2140.07	17159.59	17231.56	8.0182^**^
Reduced window	RA	0.0018	0.0011	0.0018	1431.46	11126.53	11078.49	7.7729^**^
Symmetric STDP	RA	0.0023	0.0011	0.0023	1683.24	14153.95	14066.65	8.4088
Reduced rate	IS	0.0015	0.0007	0.0014	1153.92	9159.97	9161.65	7.9382
Reduced window	IS	0.0015	0.0009	0.0015	950.02	7322.75	7318.39	7.708
Symmetric STDP	IS	0.0018	0.0013	0.0018	1211.92	7569.25	7551.88	6.2457^**^
Reduced rate	IA50	0.003	0.0007	0.003	2113.29	18946.13	19014.98	8.9652^**^
Reduced window	IA50	0.0017	0.0013	0.0017	1113.53	10964.31	10984.68	9.8465
Symmetric STDP	IA50	0.0018	0.0013	0.0018	1497.17	13115.8	13133.37	8.7604^**^
Reduced rate	IA12	0.0029	0.0009	0.0029^**^	2181.02	19180.83	19248.15	8.7944
Reduced window	IA12	0.0026	0.0013	0.0026	1603.1	13929.24	14045.97	8.6889
Symmetric STDP	IA12	0.002	0.0013	0.002	1936.32	14750.08	14709.53	7.6176^**^
**NETWORK PARAMETER VARIATIONS**								
Reduced weight	RS	0.0032^*^	0.0033^*^	0.0032^*^	3374.46	85382.1	85395.51	25.3025^*^
Asymmetric weight	RS	0.0023	0.0011	0.0023	1586.63	13208.95	13283.31	8.3252
Sparse	RS	0.0064^*^	0.0035^*^	0.0064^*^	1940.37	13669.17	13673.95	7.0446^**^
Stationary input	RS	0.0016	0.0012	0.0016	703.93	3763.03	3730.49	5.3457^**^
Reduced weight	RA	0.0033^*^	0.0047^*^	0.0033	3984.73	141100.59	140983.25	35.4103^*^
Asymmetric weight	RA	0.0029	0.0018	0.0029	2241.7	16864	16826.71	7.5229^**^
Sparse	RA	0.0058^*^	0.0038^*^	0.0058^*^	2254.03	14758.46	14768.49	6.5476^**^
Stationary input	RA	0.0015	0.0012	0.0015^**^	1092.25	6752.48	6764.09	6.1821^**^
Reduced weight	IS	0.0092^*^	0.0038^*^	0.0092^*^	7546.39	68970.17	68842.42	9.1395
Asymmetric weight	IS	0.002	0.0014	0.002	1217.88	8640.75	8645.17	7.0949^**^
Sparse	IS	0.0056^*^	0.0046^*^	0.0056^*^	1138.66	7937.2	7888.78	6.9706^**^
Stationary input	IS	0.0017	0.0012	0.0017	536.42	2427.78	2414.2	4.5259^**^
Reduced weight	IA50	0.0042^*^	0.0063^*^	0.0042^*^	4976.66	194756.25	194647.86	39.1339^*^
Asymmetric weight	IA50	0.0025	0.0016	0.0025	2229.73	18842.02	18817.85	8.4504^**^
Sparse	IA50	0.0044^*^	0.004^*^	0.0044^*^	1835.63	21273.37	21307.92	11.5892
Stationary input	IA50	0.0023	0.0016	0.0023	1319.29	10783.39	10759.42	8.1736^**^
Reduced weight	IA12	0.0038^*^	0.006^*^	0.0038	5748.78	169349.36	169372.55	29.4583^*^
Asymmetric weight	IA12	0.00338^*^	0.0013	0.0033	2697.68	18182.61	18186.25	6.7401^**^
Sparse	IA12	0.0055^*^	0.003	0.0055^*^	1328.09	16124.86	16087.73	12.1414^*^
Stationary input	IA12	0.0026	0.0016	0.0026	1627.37	12137.78	12154.85	7.4585^**^

* Indicates significant increase in value compared to the original simulations (t ≥ 3.25).

** Indicates significant decrease in value compared to original simulations (t ≤ −3.25).

Note: Triad net change, gained, and lost are reported for comparison only and were not tested for significant differences.

**Table 4:** **Core and dynamic triad characteristics for all parameter variations**.

**Condition**	**Input**	**%Remain**	**%Core**	**%Dynam**
**STDP PARAMETER VARIATIONS**				
Reduced rate	RS	48.968	69.583^*^	30.417
Reduced window	RS	45.794	68.247^*^	31.753
Symmetric STDP	RS	51.409	58.819^*^	41.181
Reduced rate	RA	53.484	63.952^*^	36.048
Reduced window	RA	51.159	60.884^*^	39.116
Symmetric STDP	RA	53.735	57.772^*^	42.228
Reduced rate	IS	44.063	75.295^*^	24.705
Reduced window	IS	44.074	70.546^*^	29.454
Symmetric STDP	IS	47.276	63.807	36.193
Reduced rate	IA50	55.817	64.173^*^	35.827
Reduced window	IA50	51.893	61.96^*^	38.04
Symmetric STDP	IA50	55.127	56.818^*^	43.182
Reduced rate	IA12	55.733	62.738^*^	37.262
Reduced window	IA12	54.32	56.463^*^	43.537
Symmetric STDP	IA12	55.933	55.349^*^	44.651
**NETWORK PARAMETER VARIATIONS**				
Reduced weight	RS	95.059	12.962^**^	87.038^*^
Asymmetric weight	RS	50.251	57.015^*^	42.985^**^
Sparse	RS	80.019	27.897^**^	72.103^*^
Stationary input	RS	36.129	82.943^*^	17.057^**^
Reduced weight	RA	99.995	3.779^**^	96.221^*^
Asymmetric weight	RA	55.443	47.616^**^	52.384^*^
Sparse	RA	79.937	25.908^**^	74.092^*^
Stationary input	RA	39.878	69.582^*^	30.418^**^
Reduced weight	IS	86.906	11.026^**^	88.974^*^
Asymmetric weight	IS	46.403	59.937^*^	40.063^**^
Sparse	IS	67.277	36.371^**^	63.629^*^
Stationary input	IS	34.629	84.903^*^	15.097^**^
Reduced weight	IA50	100	0^**^	100^*^
Asymmetric weight	IA50	56.819	47.475^*^	52.525^**^
Sparse	IA50	94.672	10.072^**^	89.928^*^
Stationary input	IA50	48.617	56.872^*^	43.128^**^
Reduced weight	IA12	100	0^**^	100^*^
Asymmetric weight	IA12	55.709	50.969^*^	49.031^**^
Sparse	IA12	83.92	23.267^**^	76.733^*^
Stationary input	IA12	49.481	57.518^*^	42.482^**^

* Indicates significant increase in value compared to original simulations (t ≥ 3.25).

** Indicates significant decrease in value compared to original simulations (t ≤ −3.25).

The total percent of triads remaining (3rd column) was not subjected to significance testing.

Overall, each of the STDP rule modifications had only marginal effects on network stability. The coefficients of variation for the number of synapses, synaptic weight, and degree were comparable to those seen in the original simulations. Nevertheless, the reduced STDP window condition, where the plasticity time constant was decreased, resulted in a significant decrease in neuronal degree variability across time when compared to the original simulations [coefficient of variation of neuronal degree; *t*_(9)_ ≤ −3.35, *p* ≤ 0.01].

The largest differences observed from the STDP modified simulations were in the percentages of core and dynamic triads. The proportion of core-to-dynamic triads was significantly increased in almost every simulation involving an STDP parameter modification [*t*_(9)_ ≥ 3.35, *p* ≤ 0.01], indicating that the composition of core and dynamic triads remaining in the network was sensitive to changes in STDP potency.

The gained-to-net ratios of triads across time were significantly reduced by the symmetric STDP condition (where potentiation and depression events have equal potency) during exposure to asynchronous input regimes (IS, IA50, IA12) and by the reduced STDP window condition during RA stimulation [*t*_(9)_ ≤ −3.35, *p* ≤ 0.01]; however, the ratios still remained high in the STDP modification conditions, suggesting that high triad turn-over is a robust effect.

Changes in network configuration parameters had more moderate effects. In the reduced synaptic weight condition, where the range of weights was reduced to half, global measures remained highly stable across time; however, significant increases in the coefficients of variation of the average weights and number of synapses were observed [*t*_(9)_ ≥ 3.35, *p* ≤ 0.01]. In every simulation involving the reduced weight condition, percentages of the core triads were reduced [*t*_(9)_ ≤ −3.35, *p* ≤ 0.01]. This effect was so great in the IA50 and IA12 simulations that no core triads were observed during the analysis interval. An effect of this parameter variation was also observed in the triad turn-over rates. This condition significantly increased gained-to-net ratios in almost all simulations [*t*_(9)_ ≥ −3.35, *p* ≤ 0.01], sometimes achieving values of over 30 triads gained for each net change in triad count. Overall, these results suggest that reducing the synaptic weight range while applying the same level of external input increased network dynamics.

Effects were mixed during the asymmetric weight condition, where inhibitory synapses were stronger on average than excitatory synapses. This variation did not significantly alter the temporal stability of any of the global measures with the exception of an increase in the coefficient of variation of the number of synapses during the IA12 input regime [*t*_(9)_ ≥ 3.35, *p* ≤ 0.01]. Additionally, the core-to-dynamic triad ratio was significantly reduced following RA input [*t*_(9)_ ≤ −3.35, *p* ≤ 0.01]; however, all other input types resulted in increases in the ratio [*t*_(9)_ ≥ 3.35, *p* ≤ 0.01]. The gained-to-net ratio of triads decreased in this network in every input regime except during RS input. Nevertheless, values remained high, suggesting that substantial triad turn-over continued to occur.

Reducing network connectivity (sparse connectivity condition) also resulted in several significant differences. Although they remained low, this condition increased the coefficients of variation of all of the global measures in almost all of the simulations [*t*_(9)_ ≥ 3.35, *p* ≤ 0.01]. This condition also significantly decreased the core-to-dynamic triad ratios in every simulation [*t*_(9)_ ≤ −3.35, *p* ≤ 0.01]. Effects of sparse connectivity on gained-to-net ratios varied according to input regime: RA, RS, and IS input decreased the ratio while IA12 input increased the ratio [*t*_(9)_ ≤ −3.35; *t*_(9)_ ≥ 3.35; *p* ≤ 0.01, all]. Similar to reducing the synaptic weight range, reducing the number of initial synapses seemed to increase network dynamics.

During the stationary input condition, where the same set of neurons received all external input, global stability was not significantly altered except for an increase in the stability of the average neuronal degree across time [i.e., decreased coefficient of variation of degree; *t*_(9)_ ≤ −3.35, *p* ≤ 0.01]. This condition also significantly increased the core-to-dynamic triad ratios in all simulations [*t*_(9)_ ≥ 3.35, *p* ≤ 0.01]. The gained-to-net triad change ratio was also decreased in every simulation [*t*_(9)_ ≤ −3.35, *p* ≤ 0.01]. These results suggest that applying external input to a fixed input layer increases network structural stability.

## Discussion

Previous theoretical and empirical work has demonstrated that STDP is capable of selecting unique and presumably advantageous topological features, such as small-world properties and specific mosaics of motifs (Shin and Kim, 2006; Ren et al., 2009). This report demonstrates that not only does STDP select these features, but it also maintains them across a broad range of input regimes and parameter values. The low variability in measures such as synapse count, clustering, degree distributions and frequencies of motif types across time reveals that STDP-driven networks are highly stable even in the presence of noisy and unpredictable input. However, this global stability belies a dynamic local topology which remains flexible and responsive. This balance of stability and flexibility is critical for unsupervised learning and underscores the viability of STDP as a powerful tool not only during neurobiological development but throughout the lifespan.

The main finding of this study is that networks undergoing continual STDP evolve globally stable topologies while the local topology remains highly dynamic, as evidenced by the continual emergence, disappearance, and reshaping of individual three-neuron triads. This finding is consistent with several recent studies where local cortical activity was monitored over long durations while adult mice underwent learning. For example, Huber and colleagues (2012) recently reported changes in primary motor cortex while mice learned an object detection task. The authors found that as the mice learned the task, population-level representations stabilized despite an on-going instability of representations in individual neurons. It was also recently reported that, following whisker-trimming, overall population activity in the barrel cortex of adult mice re-stabilized; however, there was a radical shifting of activity in individual neurons. Previously silent neurons increased their responsiveness to spared-whisker stimulation, while the formerly most active neurons decreased their responsiveness (Margolis et al., 2012). These studies did not specifically investigate the synaptic plasticity mechanisms responsible for these changes; however, in the present study it was found that STDP similarly maintains global stability in the presence of active local dynamics, and therefore may play a role in the rodent cortical plasticity that was observed during learning.

The balance between stable and dynamic network structure is emphasized by the discovery of both persistent core triads and transient dynamic triads. Core triads were composed of strong and stable synapses, similar to patterns that have been observed *in vitro* and *in vivo*. Song et al. (2005) found a core network of strongly connected triads among layer V neurons in rat visual cortex. The authors suggest that these triads drive network activity and are responsible for the stereotypical firing patterns observed in cortical slices. Consistent with this hypothesis, Lefort and colleagues (2009) have reported the presence of sparse, strong and reliable synapses in rodent somatosensory cortical columns. In a model, they found that minimal synchronous input was capable of driving networks that possessed these synapses. Interestingly, in the present study, synchronous input increased the presence and strength of core triads. STDP appears to act as a self-organizing mechanism that leads to the emergence of greater or fewer core triads depending upon external input. If these core triads drive the dominant patterns of network activity as suggested, this may explain why qualitatively different input characteristics cause distinct patterns of population activity like those seen in Figure 2.

Asynchronous input, on the other hand, resulted in an increase in dynamic triads. Asynchronous cortical activity is a hallmark feature of awake, adult mammals. The presence of desynchronization appears to emerge during perinatal neocortical development and emerges largely independently of external stimulation (Golshani et al., 2009; Rochefort et al., 2009). This Spontaneous desynchronized activity may lead to a ubiquity of dynamic triads as networks become more refined. The presence of dynamic triads in these networks may have important functional consequences. After exploring a range of brain and neural networks from several species, Sporns and Kotter (2004) proposed a distinction between structural and functional motifs (triads). According to their interpretation, the physical connections between neurons or brain regions form structural motifs and functional motifs are transient activations upon these structural motifs recruited during on-going information processing. Some structural motifs possess a repertoire of functional motifs because distinct subsets of connections can be selectively activated. Motif types composed of more connections possess larger repertoires because of the number of functional motifs that can be formed from them. The authors have argued that topologies comprised of a small set of structural motifs with large repertoires of functional motifs are highly efficient because they increase computational capacity with low wiring costs. In the present report, dynamic triads are analogous to the functional motifs that Sporns and Kotter describe. Their presence illustrates how a simple and biologically realistic mechanism, STDP, provides a viable means for recruiting functional motifs to accommodate on-going demands. Further, the set of the functional motifs which are employed at a given moment can be modified by synaptic potentiations and depressions. Since a single synapse invariably participates in a multitude of motifs, subtle changes in the weights of just a few synapses can lead to network-wide changes in functional motif activity. Thus, STDP increases network efficiency even further by adding another dimension to computational capacity (the mosaic of active functional motifs) with relatively low metabolic costs.

A novel and unexpected finding in this study was the degree of “small-worldness” observed during on-going STDP. The observation of increased neural clustering coupled with increased path lengths between neurons suggests a much more localized network than would be expected in a small-world topology. However, the highly dynamic nature of the path lengths during the simulations must be considered. Widely fluctuating path distances between neurons during the simulations suggest that there were moments of greater small-worldness intermixed with moments of more localization and restriction. In this study, distance is not merely interpreted by the number of synapses between neurons but also reflects the strengths of the synapses separating them. In this case, a path formed by several strong synapses may be more advantageous than a path consisting of a single weak synapse. Again, STDP may enhance the functional capacity of these networks by varying the strengths of key synapses rather than through forming network-wide changes. The modulating path lengths may act as a functional gating mechanism which could significantly enhance the computational properties of the networks. Incorporating synaptic weights and directionality into the evaluation of path lengths may also explain why this study did not find the emergence of a consistent small-world topology while similar studies did (Suzuki and Ikeguchi, 2005; Shin and Kim, 2006). Indeed, when path lengths were evaluated without consideration of direction or weight in the present simulations, values dropped considerably and practically no temporal variability was observed (data not shown). The potential flexibility of small-worldness and the qualitatively different results that are observed when directionality and synaptic strength are accounted for warrant further investigation in both model and biological systems.

One of the important conclusions that may be drawn from this report is that complex networks can appear static at one level of analysis and yet be highly dynamic at another level. The explorations reported here provide some insight into how to assess evolving neural networks and other types of networks which change over time. It appears that an important and thus far overlooked metric is motif turn-over rates, and perhaps the turn-over rates of other network constituents. The highly dynamic behavior and shifting participation of individual triads suggests that they may play an important computational role in the networks. As a caveat, researchers should be cautious in assessing the activity of any complex network without taking into account the unique participations of individual network constituents.

This work presents novel findings regarding the influence of STDP on evolving network topology; however, a number of outstanding questions remain. Inhibitory synapses were not modified in these simulations, although it is likely that these synapses change the functionality of triads (Li, 2008). What influence would inhibitory plasticity have? The spike-timing mechanisms at these synapses appear to be governed by different rules than at excitatory synapses (Haas et al., 2006), and unique behaviors occur in predominantly inhibitory networks where STDP is at play (Fino and Venance, 2010; Fino et al., 2010). Further, STDP represents only one form of synaptic modification, and indeed only one type of Hebbian plasticity. We selected STDP as the candidate plasticity mechanism in our simulations based on previous work which has shown that STDP can promote stable activity and reduce noise in recurrent networks (Diesmann et al., 1999; Bohte and Mozer, 2007; Takahashi et al., 2009). While the results here suggest that STDP plays the key role in stable yet flexible network self-organization, other forms of plasticity may lead to similar network topologies or wholly unique yet viable topologies of their own. It should be noted that we experimented with a Hebbian plasticity model based on correlations between pre-synaptic and post-synaptic firing rates. The model led to completely different behavior, including a total absence of dynamic triads early in the simulations (unpublished data). These preliminary findings suggest that direct comparisons between the effects of STDP and other plasticity models on topological evolution warrants further investigation. The inclusion of other plastic changes such as the formation of new synapses, on-going synaptic decay, and the role of neuromodulators undoubtedly add to the complexity of topological dynamics, as well. Another potential limitation is the time-scale of synaptic modification studied. While these simulations represented 2 h of activity in small networks, the persistence of the stable topological features observed such as core triads may erode at longer timescales and more transient structural fluctuations may have been missed due to sampling rate limitations. Finally, present study only focused on three-neuron motifs as the main feature of local topology. We selected three-neuron motifs for comparison to previous work and because of their computational and analytical tractability; however, motifs of other sizes undoubtedly possess additional unique responses to on-going STDP that remain to be characterized. Further theoretical work coupled with empirical explorations will provide a more complete picture of the on-going topological dynamics of neural networks and the factors that influence it.

## Conclusion

Over the past decade, network science has provided a number of insights into an array of complex systems such as social, biological, and technological networks, as well as real and modeled neural and cortical networks. As a consequence, network scientists have developed new ways of approaching complex systems and have uncovered a number of common features shared by many different types of systems. Nevertheless, despite general agreement that the topology of many of these systems continually evolves, only a handful of studies have begun to explore their on-going structural dynamics. New measures and methodologies are being developed to capture the unique properties of evolving graphs (e.g., Acer et al., 2011; Starnini et al., 2012), and a better understanding of the temporal characteristics of specific systems, such as human contact networks and technological networks, is beginning to emerge (Scherrer et al., 2008; Kim et al., 2012). However, to date, research into the on-going topological dynamics of neural and cortical networks is almost non-existent. In addition, although there is a spate of theoretical and empirical work addressing the functionality of STDP, the continuing role it plays in shaping network organization beyond early development is underexplored [notable exceptions include recent work by Gambino and Holtmaat (2012) and Pawlak et al. (2013)]. The present work sheds light onto the evolving topology of neural networks and the role that STDP plays in shaping and maintaining this topology. It remains to be seen whether the discoveries made here are unique to STDP-driven neural networks or whether they represent general features of broader classes of complex systems.

### Conflict of interest statement

The authors declare that the research was conducted in the absence of any commercial or financial relationships that could be construed as a potential conflict of interest.
